# Theory of Planned Behaviour Constructs as Predictors of Antiplatelet Medication Adherence Following Percutaneous Coronary Intervention: A Cross-Sectional Study in Saudi Arabia

**DOI:** 10.3390/healthcare14060811

**Published:** 2026-03-22

**Authors:** Muteb Aljuhani, Asrar S. Almutairi, Waleed M. Alshehri, Abdulaziz M. Alodhailah

**Affiliations:** 1 Department of Community Health, Mental and Psychiatric Nursing, Imam Mohammad Ibn Saud Islamic University (IMSIU), Riyadh 11564, Saudi Arabia; 2 Community and Psychiatric Mental Health Nursing Department, College of Nursing, Princess Nourah bint Abdulrahman University, Riyadh 11671, Saudi Arabia; 3 Department of Medical-Surgical Nursing, College of Nursing, King Saud University, Riyadh 11451, Saudi Arabia

**Keywords:** Theory of Planned Behaviour, medication adherence, antiplatelet therapy, percutaneous coronary intervention, behavioural intention, Saudi Arabia

## Abstract

**Background**: Theoretical frameworks are essential for understanding and predicting medication adherence behaviours. The Theory of Planned Behaviour (TPB) posits that behavioural intentions, shaped by attitudes, subjective norms, and perceived behavioural control, are the proximal determinants of behaviour. This cross-sectional study examined associations between TPB constructs and antiplatelet medication adherence among Saudi patients following percutaneous coronary intervention (PCI). **Methods**: A cross-sectional survey was conducted among 236 Saudi adults post-PCI at two tertiary cardiac centres in Riyadh. TPB constructs (attitude, subjective norms, perceived behavioural control, intention) were assessed using a validated questionnaire. Adherence was measured via the Morisky Medication Adherence Scale-8 (MMAS-8). Hierarchical multiple regression examined associations between TPB constructs and adherence, controlling for demographic and clinical variables. **Results**: The results demonstrated significant associations with adherence. In the final regression model, intention (β = 0.273, *p* < 0.001), perceived behavioural control (β = 0.189, *p* = 0.007), and subjective norms (β = 0.142, *p* = 0.038) were significantly associated with adherence. Attitude was not significantly associated (β = 0.087, *p* = 0.194). The TPB constructs explained an additional 18.7% of variance in adherence beyond demographic and clinical factors. **Conclusions**: The TPB provides a useful framework for understanding antiplatelet adherence patterns in Saudi post-PCI patients. These findings suggest that interventions addressing behavioural intentions, perceived control over medication-taking, and normative influences from significant others may potentially enhance adherence outcomes. Theory-informed nurse-led interventions incorporating strategies such as implementation intentions and family involvement are recommended.

## 1. Introduction

Medication adherence is a complex behaviour determined by psychological, social, and contextual factors. Theoretical frameworks provide structured approaches to identify modifiable factors [1] that can be targeted through evidence-based interventions [2]. Among available theories, the Theory of Planned Behaviour (TPB) has been widely applied to predict and explain health behaviours, including medication adherence across chronic conditions [3,4].

The TPB, developed by Icek Ajzen, proposes that behaviour is determined by behavioural intention, which in turn is shaped by three constructs: attitude toward the behaviour, subjective norms, and perceived behavioural control [5]. Attitude refers to an individual’s positive or negative evaluation of performing a behaviour, in this context, beliefs about the benefits and drawbacks of taking antiplatelet medication. Subjective norms reflect perceived social pressure from important others (family, healthcare providers) to perform or not perform the behaviour. Perceived behavioural control encompasses beliefs about one’s capability to perform the behaviour, including perceptions of facilitating factors and barriers [6].

The TPB has been successfully applied to understand medication adherence across various populations and conditions, including hypertension, diabetes, and cardiovascular disease [7,8]. Studies consistently demonstrate that intention is a strong proximal predictor of adherence, while the relative contributions of attitude, subjective norms, and perceived behavioural control vary across contexts [9]. Perceived behavioural control has emerged as particularly important for adherence behaviours, as practical barriers, such as forgetfulness, medication complexity, and side effects, can impede even highly motivated individuals from consistent medication-taking [10].

Following percutaneous coronary intervention (PCI), adherence to dual antiplatelet therapy (DAPT) is essential for preventing stent thrombosis and recurrent cardiovascular events [11]. Non-adherence to antiplatelet medications substantially increases the risk of adverse outcomes, including myocardial infarction and death [12]. Despite this clinical importance, adherence rates remain suboptimal globally, with estimates suggesting that 20–50% of patients do not take medications as prescribed [13].

In Saudi Arabia, where cardiovascular disease represents a leading cause of mortality, understanding determinants of antiplatelet adherence is particularly important [14]. Cultural factors may uniquely shape TPB constructs in this context. Subjective norms, for example, may be particularly influential given the prominent role of family in Saudi healthcare decision-making and the cultural emphasis on collective responsibility for health [15]. Religious beliefs, including concepts of divine predestination and trust in God’s will, may influence attitudes toward medication-taking and perceived control over health outcomes [16]. These cultural considerations underscore the importance of empirically examining TPB constructs within the Saudi population rather than assuming generalizability of findings from Western contexts.

Nurses play a central role in promoting medication adherence through patient education, counselling, and follow-up support [17]. Theory-based interventions have demonstrated greater effectiveness than atheoretical approaches, as they enable systematic targeting of identified determinants [18]. Understanding which TPB constructs most strongly predict adherence in Saudi post-PCI patients can guide nurses in designing culturally appropriate, theory-informed interventions with optimal potential for behaviour change.

### Aims of the Study

This study aimed to:

Describe levels of TPB constructs (attitude, subjective norms, perceived behavioural control, intention) among Saudi patients following PCI.

Examine associations between TPB constructs and antiplatelet medication adherence.

Identify which TPB constructs most strongly predict adherence to inform theory-based nursing interventions.

## 2. Materials and Methods

### 2.1. Study Design

A cross-sectional quantitative survey design was employed to examine TPB constructs as predictors of antiplatelet medication adherence among post-PCI patients in Saudi Arabia.

### 2.2. Theoretical Framework

The TPB served as the guiding theoretical framework [5]. The theory posits that behavioural intention is the most proximal predictor of behaviour, determined by three conceptually independent constructs:

Attitude toward the behaviour: Beliefs about the outcomes of medication-taking and evaluations of those outcomes.

Subjective norms: Perceived expectations of important referents (family, healthcare providers) regarding medication-taking.

Perceived behavioural control: Beliefs about factors that facilitate or impede medication-taking and perceived power over those factors.

According to the TPB, individuals are more likely to adhere to medication regimens when they hold positive attitudes, perceive social pressure to adhere, believe they have control over adherence behaviour, and form strong intentions to take medications as prescribed.

### 2.3. Setting and Sample

The study was conducted at two tertiary cardiac referral centres in Riyadh, Saudi Arabia. The sample comprised 236 Saudi adults (≥18 years) who had undergone PCI within the preceding 12 months and were prescribed antiplatelet therapy. Patients with cognitive impairment, severe psychiatric illness, or inability to provide consent were excluded. Purposive sampling during routine cardiology follow-up visits yielded a response rate of 78.1%.

### 2.4. Sample Size Determination

Sample size was calculated based on multiple regression analysis requirements. For detecting medium effect sizes with eight predictor variables (four TPB constructs plus four demographic/clinical covariates), a minimum sample of 107 participants was required at 80% power and α = 0.05 [19]. The achieved sample of 236 participants exceeded this requirement, providing adequate statistical power.

### 2.5. Data Collection Instruments

Demographic and Clinical Profile: Items assessed age, gender, marital status, educational attainment, employment status, monthly income, comorbidities (heart disease, hypertension, diabetes mellitus, kidney disease), time since heart disease diagnosis, and number of previous PCIs.

The TPB questionnaire items were developed using established TPB protocol guidelines (Ajzen’s standard operationalization approach) and systematically adapted to the Saudi Arabian cultural context. To ensure cultural and linguistic appropriateness:

Translation Process: A forward-backward translation procedure was employed. Two independent bilingual medical translators (Arabic-English) first translated items from English to Arabic. A third bilingual expert then performed independent back-translation to English. Any discrepancies were resolved through consensus discussion to ensure semantic equivalence while maintaining cultural relevance.

Cognitive Testing: Cognitive interview testing was conducted with 15 pilot participants (ages 45–75, representing the target population but not included in the main study). Participants were asked to comment on item clarity, comprehensibility, and cultural appropriateness. Minor wording adjustments were implemented based on cognitive testing feedback to enhance Saudi cultural fit.

Construct Validity: Exploratory factor analysis (EFA) using principal axis factoring with promax rotation was conducted to validate the theoretical four-factor structure of the TPB. Results confirmed the expected structure with factor loadings > 0.50 for all items.

Administration: The questionnaire was available in both Arabic (primary) and English (for participants preferring English) versions.

Internal Consistency: Cronbach’s alpha coefficients were calculated for each subscale (attitude α = 0.78; subjective norms α = 0.81; perceived behavioural control α = 0.76; intention α = 0.84), all demonstrating acceptable reliability (α > 0.75). The TPB questionnaire assessed the four key constructs:

Attitude (6 items): Assessed beliefs about outcomes and evaluations of antiplatelet medication-taking (e.g., “Taking my antiplatelet medication helps prevent another heart attack”; “Taking my antiplatelet medication is beneficial for my health”). Responses were recorded on a 5-point Likert scale (1 = strongly disagree to 5 = strongly agree).

Subjective Norms (4 items): Assessed perceived expectations from significant others (e.g., “My family thinks I should take my antiplatelet medication as prescribed”; “My doctor expects me to take my medication regularly”). Responses on 5-point Likert scale.

Perceived Behavioural Control (5 items): Assessed beliefs about facilitating factors and barriers to medication-taking (e.g., “I am confident I can remember to take my medication”; “Taking my medication regularly is entirely up to me”). Responses on 5-point Likert scale.

Intention (3 items): Assessed strength of intention to adhere to antiplatelet therapy (e.g., “I intend to take my antiplatelet medication exactly as prescribed”; “I plan to continue taking my medication for as long as recommended”). Responses on 5-point Likert scale.

Construct scores were calculated as mean item scores, with higher scores indicating more positive attitudes, stronger perceived norms, greater perceived control, and stronger intentions. The questionnaire demonstrated acceptable internal consistency in this sample (Cronbach’s α: attitude = 0.78, subjective norms = 0.81, perceived behavioural control = 0.76, intention = 0.84).

Morisky Medication Adherence Scale-8 (MMAS-8): This validated 8-item instrument measured self-reported adherence to antiplatelet therapy [20]. The MMAS-8 comprises seven yes/no dichotomous items and one item with a 5-point Likert response scale. The 8th item was scored using fractional values (0, 0.25, 0.5, 0.75, 1.0) to maintain a continuous 0–8 total scale, with higher scores indicating better adherence. For regression analyses, MMAS-8 scores were treated as a continuous outcome variable. The scale demonstrated acceptable internal consistency in this sample (Cronbach’s α = 0.78).

### 2.6. Data Collection Procedure

Eligible participants were defined as Saudi adults aged ≥18 years who had undergone percutaneous coronary intervention within the preceding 12 months and were currently prescribed dual or single antiplatelet therapy. Exclusion criteria included: (1) cognitive impairment or inability to complete questionnaires independently, (2) severe psychiatric illness requiring acute hospitalization, (3) inability to provide informed consent, or (4) non-Arabic/non-English speaking status.

Recruitment occurred during routine cardiology follow-up visits at two tertiary cardiac centres in Riyadh. The researcher systematically approached consecutive eligible patients during clinic hours. Of 303 patients approached, 236 completed questionnaires (response rate = 77.9%), with 67 declining participation (refusal rate = 22.1%). Reasons for non-participation included: time constraints (n = 34), lack of interest (n = 18), and privacy concerns (n = 15).

Questionnaires were administered in private consultation rooms with options for: (1) independent self-completion, or (2) assisted completion by research assistants for patients with literacy limitations or vision impairment. Survey completion required approximately 15–20 min.

### 2.7. Data Analysis

Descriptive statistics summarized TPB construct scores (means, standard deviations) and adherence levels (frequencies, percentages). Pearson correlation coefficients examined bivariate associations among TPB constructs and with adherence scores.

Hierarchical multiple regression examined TPB constructs as predictors of adherence (continuous MMAS-8 scores). In Step 1, demographic and clinical covariates (age, gender, education, and comorbidity count) were entered. In Step 2, TPB constructs (attitude, subjective norms, perceived behavioural control, intention) were added.

The incremental variance explained by TPB constructs (18.7% beyond demographics) is consistent with effect sizes typically reported in TPB health behaviour research meta-analyses (R^2^ = 0.15–0.25 in cross-sectional studies). This suggests that the magnitude of associations observed in this Saudi population aligns with international TPB literature.

Regression assumptions were evaluated, including multicollinearity (VIFs), normality of residuals (Q-Q plots), and homoscedasticity (visual inspection of residual plots), with no significant violations detected. Statistical significance was set at *p* < 0.05. Analyses were performed using SPSS version 26.

### 2.8. Ethical Considerations

Ethical approval was obtained from both participating institutions (IRB No. 17/0174/IRB and IRB No. 17-019E). The study adhered to Declaration of Helsinki principles. Participants provided written informed consent and could withdraw at any time without consequence.

## 3. Results

### 3.1. Sample Characteristics

The sample (n = 236) was predominantly male (73.7%) with a mean age of 56.8 years (SD = 11.3). Most participants were married (80.9%) and had limited formal education (33.1% no schooling, 45.3% general education). Comorbidities were highly prevalent: pre-existing heart disease (69.5%), hypertension (51.3%), and diabetes mellitus (45.0%).

### 3.2. Theory of Planned Behaviour Construct Scores

Descriptive statistics for TPB constructs are presented in Table 1. Intention demonstrated the highest mean score (M = 4.21, SD = 0.73), indicating strong intentions to adhere among most participants. Attitude scores were also relatively high (M = 4.08, SD = 0.69), reflecting generally positive beliefs about antiplatelet medication benefits. Subjective norms showed moderate-to-high scores (M = 3.89, SD = 0.82), suggesting perceived social support for adherence. Perceived behavioural control exhibited the greatest variability (M = 3.62, SD = 0.91), with scores spanning the full range, indicating heterogeneous beliefs about medication-taking capability.

### 3.3. Medication Adherence

The mean MMAS-8 score was 5.12 (SD = 1.87). Categorical classification revealed that 55.4% of participants demonstrated low adherence (score < 6), 34.8% medium adherence (score 6–7), and 9.9% high adherence (score = 8).

### 3.4. Correlations Among TPB Constructs and Adherence

Pearson correlations are presented in Table 2. All TPB constructs were significantly positively correlated with each other and with adherence. Intention showed the strongest correlation with adherence (r = 0.412, *p* < 0.001), followed by perceived behavioural control (r = 0.378, *p* < 0.001), subjective norms (r = 0.298, *p* < 0.001), and attitude (r = 0.246, *p* < 0.001).

Intercorrelations among TPB constructs were moderate, indicating related but distinct constructs. Variance inflation factors ranged from 1.28 to 1.67, indicating no problematic multicollinearity.

### 3.5. Hierarchical Regression Analysis

Hierarchical multiple regression results are presented in Table 3. In Step 1, demographic and clinical variables (age, gender, education, comorbidity count) explained 8.4% of variance in adherence (R^2^ = 0.084, F (4, 231) = 5.31, *p* < 0.001). Education was the only significant demographic predictor (β = 0.198, *p* = 0.003).

In Step 2, adding TPB constructs significantly improved model fit (ΔR^2^ = 0.187, ΔF (4, 227) = 14.82, *p* < 0.001), with the final model explaining 27.1% of the total variance (R^2^ = 0.271, F (8, 227) = 10.56, *p* < 0.001).

In the final model, three TPB constructs were significantly associated with adherence: intention (β = 0.273, *p* < 0.001), perceived behavioural control (β = 0.189, *p* = 0.007), and subjective norms (β = 0.142, *p* = 0.038). Attitude was not significantly associated with adherence (β = 0.087, *p* = 0.194). Education remained significant in the final model (β = 0.132, *p* = 0.041).

In the final model, three TPB constructs emerged as significant independent predictors of adherence:

Intention was the strongest predictor (β = 0.273, *p* < 0.001) (small-to-medium effect). Participants with stronger intentions to adhere demonstrated significantly better adherence behaviour.

Perceived Behavioural Control was the second strongest predictor (β = 0.189, *p* = 0.007) (small effect). Greater perceived control over medication-taking was associated with better adherence.

Subjective Norms significantly predicted adherence (β = 0.142, *p* = 0.038) (small effect). Stronger perceived social expectations for adherence were associated with better medication-taking behaviour.

Attitude was not a significant independent predictor after controlling for other TPB constructs (β = 0.087, *p* = 0.194).

Education remained a significant predictor in the final model (β = 0.132, *p* = 0.041), though its effect was attenuated compared to Step 1, suggesting partial mediation through TPB constructs.

## 4. Discussion

This study provides the first comprehensive examination of TPB constructs as predictors of antiplatelet medication adherence among Saudi post-PCI patients. The findings demonstrate that the TPB provides a useful framework for understanding adherence behaviour in this population, with intention, perceived behavioural control, and subjective norms emerging as significant independent predictors. These results have important implications for the design of theory-based nursing interventions to enhance medication adherence.

Consistent with TPB theoretical propositions and prior literature, intention demonstrated the strongest association with adherence [21,22]. Patients with strong intentions to take their antiplatelet medications as prescribed reported significantly better adherence, consistent with the TPB framework. This finding underscores the importance of fostering strong behavioural intentions as a key intervention target. However, the intention-behaviour gap—the observation that intentions do not always translate into action—suggests that additional strategies are needed to support behaviour enactment [23].

Perceived behavioural control demonstrated the second strongest association with adherence, consistent with meta-analytic evidence on the importance of perceived control for adherence behaviours. Patients who reported greater perceived control over their medication-taking reported better adherence. These findings suggest potential clinical implications for future interventions. Nursing interventions should address practical barriers to medication-taking and enhance patients’ confidence in their ability to overcome challenges [24]. Strategies such as simplifying regimens, providing pill organizers, establishing medication-taking cues linked to daily routines, and problem-solving around anticipated barriers may strengthen perceived behavioural control [25].

The significant role of subjective norms is particularly noteworthy in the Saudi cultural context. Family involvement in healthcare is a prominent feature of Saudi society, with spouses, adult children, and extended family members often participating in medical consultations and home care [26]. The finding that perceived social expectations from family and healthcare providers independently predict adherence suggests that interventions should actively engage significant others. Family-based educational interventions, in which spouses or caregivers receive information about medication importance and are encouraged to provide reminders and support, may be particularly effective in this cultural setting [17].

The significant association of subjective norms (β = 0.142, *p* = 0.038) is particularly noteworthy in the Saudi cultural context, where extended family structures and collective healthcare decision-making are normative [26]. Our finding that perceived social expectations from family and healthcare providers independently predict antiplatelet adherence aligns with broader research on social determinants of health behaviours in collectivist cultures (DiMatteo, 2004) [27]. Moreover, the cultural emphasis on family responsibility for health outcomes in Saudi society—reflected in high family involvement in medical consultations and home-based care—suggests that family-centred interventions may achieve greater effectiveness than individualistic approaches common in Western contexts. Family-based educational strategies, in which spouses or caregivers receive explicit information about medication importance and are trained as adherence reminders and supporters, represent theoretically informed adaptations suited to this cultural context [17].

The non-significance of attitude as an independent predictor, despite its bivariate correlation with adherence (r = 0.246, *p* < 0.001), is consistent with some prior TPB research in medication adherence contexts [27]. This pattern may reflect several possible mechanisms: (1) Relative homogeneity of attitudes in this sample, with most participants (M = 4.08/5.0) holding strong positive beliefs about antiplatelet medication benefits, limiting the construct’s discriminative capacity; (2) Mediation of attitude effects through intention, with the direct effect of attitude on behaviour becoming minimal once intention is controlled [28]; and (3) The context-specific importance of volitional control and social influences over individual attitudes in Saudi healthcare decision-making, where family and healthcare provider expectations may supersede personal evaluative beliefs.

The incremental variance explained by TPB constructs (18.7% beyond demographics) is consistent with effect sizes typically observed in TPB health behaviour research [29]. While substantial variance remains unexplained, reflecting the multifactorial nature of adherence, the identified predictors represent viable intervention targets. Unmeasured factors potentially contributing to adherence include medication side effects, health literacy, healthcare system factors, and disease-specific variables not captured in this study [30].

### 4.1. Implications for Nursing Practice

The findings support several theory-based recommendations for nursing practice:

1. Intention-Enhancement Strategies: Nurses should employ techniques that strengthen patients’ intentions to adhere, such as eliciting verbal commitments to take medications, setting specific adherence goals, and using implementation intentions, such as plans specifying when, where, and how medications will be taken (e.g., “I will take my Plavix with breakfast at 7 am every morning”) [3,31].
2. Perceived Control Enhancement: Nursing interventions should systematically address barriers to medication-taking. This includes practical strategies (pill organizers, reminder systems, routine integration), self-efficacy building through mastery experiences, and problem-solving around anticipated challenges such as travel, fasting, or schedule disruptions.
3. Normative Influence Activation: Given the significance of subjective norms, nurses should involve family members in medication education and encourage their active support role. Healthcare provider expectations should be clearly communicated—nurses and physicians should explicitly convey that they expect patients to take medications as prescribed, as this is important for cardiac health.
4. Tailored Interventions: Assessment of TPB constructs can enable personalized intervention targeting. Patients with low perceived control may benefit from barrier-focused counselling, while those with weak normative influence may benefit from family involvement strategies.

### 4.2. Strengths and Limitations

This study contributes to theory application in Middle Eastern cardiovascular populations, addressing a significant gap in the literature. The use of a validated theoretical framework enables evidence-based intervention development. Hierarchical regression methodology appropriately controlled for demographic confounders.

Several limitations warrant acknowledgment. The cross-sectional design precludes causal inference; prospective studies are needed to confirm predictive relationships. Self-report measures may be subject to social desirability and recall biases, potentially leading to overestimation of adherence rates. The true prevalence of medication non-adherence in this population may exceed the 55.4% we observed. The TPB questionnaire, while demonstrating acceptable reliability, would benefit from further validation in Saudi Arabia. The sample was recruited from two urban tertiary centres, potentially limiting generalizability. Future studies should employ multiple adherence measurement methods, including objective measures such as pharmacy refill data, electronic medication dispensers, and biomarker assessments (e.g., platelet function tests), to triangulate self-report findings and reduce bias.

The final model explained 27.1% of adherence variance, leaving 72.9% unexplained. Unmeasured factors potentially contributing to this unexplained variance include health literacy, psychological factors (depression, anxiety, psychological distress), medication side effects and adverse effects, healthcare system factors (medication accessibility, cost, pharmacy dispensing practices), and disease-specific variables (time since diagnosis, disease severity, comorbidity burden). Future research should prospectively examine these variables to develop more comprehensive models of antiplatelet adherence in Saudi populations.

## 5. Conclusions

This study demonstrates that the Theory of Planned Behaviour provides a useful framework for understanding antiplatelet medication adherence among Saudi post-PCI patients. Intention, perceived behavioural control, and subjective norms emerged as significant independent predictors of adherence. The findings suggest that theory-informed interventions targeting behavioural intentions, perceived control over medication-taking, and normative influences from family members and healthcare providers may potentially enhance adherence in similar populations. Nursing interventions incorporating culturally tailored strategies such as implementation intentions, family education programmes, and barrier-reduction counselling warrant examination in future prospective or intervention studies to confirm their effectiveness.

## Figures and Tables

**Table 1:** Theory of Planned Behaviour construct scores (n = 236).

Construct	Mean	SD	Range	Possible Range
Attitude	4.08	0.69	1.83–5.00	1–5
Subjective Norms	3.89	0.82	1.25–5.00	1–5
Perceived Behavioural Control	3.62	0.91	1.20–5.00	1–5
Intention	4.21	0.73	1.67–5.00	1–5

**Table 2:** Pearson correlations among TPB constructs and adherence (n = 236).

Variable	1	2	3	4	5
1. Attitude	-				
2. Subjective Norms	0.387 ***	-			
3. PBC	0.312 ***	0.428 ***	-		
4. Intention	0.456 ***	0.489 ***	0.521 ***	-	
5. Adherence (MMAS-8)	0.246 ***	0.298 ***	0.378 ***	0.412 ***	-

Note. PBC = Perceived Behavioural Control. *** *p* < 0.001.

**Table 3:** Hierarchical multiple regression: TPB predictors of antiplatelet adherence.

Variable	Step 1 β	Step 2 β	*p* (Step 2)
Step 1: Demographics/Clinical			
Age	0.089	0.056	0.362
Gender (male)	0.067	0.043	0.478
Education	0.198 **	0.132 *	0.041
Comorbidity Count	−0.112	−0.078	0.198
Step 2: TPB Constructs			
Attitude	—	0.087	0.194
Subjective Norms	—	0.142 *	0.038
Perceived Behavioural Control	—	0.189 **	0.007
Intention	—	0.273 ***	<0.001
R^2^	0.084	0.271	
ΔR^2^	—	0.187 ***	
F	5.31 ***	10.56 ***	

Note. * *p* < 0.05, ** *p* < 0.01, *** *p* < 0.001.

## Data Availability

The datasets generated and/or analyzed during the current study are available from the corresponding author on reasonable request. Due to privacy restrictions, data cannot be shared publicly.
